# Experimental research of host macrophage canceration induced by glioma stem progenitor cells

**DOI:** 10.3892/mmr.2014.3032

**Published:** 2014-12-02

**Authors:** AIDONG WANG, XINGLIANG DAI, BAOQIAN CUI, XIFENG FEI, YANMING CHEN, JINSHI ZHANG, QUANBIN ZHANG, YAODONG ZHAO, ZHIMIN WANG, HUA CHEN, QING LAN, JUN DONG, QIANG HUANG

**Affiliations:** 1 Department of Neurosurgery, The Second Affiliated Hospital of Soochow University, Suzhou, Jiangsu 215004, P.R. China; 2 Department of Neurosurgery, Suzhou Kowloon Hospital, School of Medicine, Shanghai Jiaotong University, Suzhou, Jiangsu 215021, P.R. China; 3 Department of Neurosurgery, Shanghai 10th People’s Hospital, Shanghai Tongji University, Shanghai, Jiangsu 200072, P.R. China; 4 Department of Neurosurgery, Nanjing First Hospital, Nanjing Medical University, Nanjing, Jiangsu 210006, P.R. China

**Keywords:** tumor transplantation, fluorescent nude mice, macrophage, cell canceration

## Abstract

The involvement of tumor-associated macrophages in tumor progression is an indisputable fact. However, whether the growth-promotion effects of macrophages towards tumors in the aggressive stage affect their own canceration remains unknown. In the present study, human glioma stem/progenitor cells transfected with red fluorescent protein gene (SU3-RFP) were seeded inside the abdominal cavity of transgenic nude mice, of which all nucleated cells could express green fluorescent protein (GFP), forming a tumor model with a double-color RFP/GFP fluorescent tracer. Ascites and tumor nodules from tumor-bearing mice were cultured, then the GFP^+^ cells were separated for clonal culture and further related phenotypic characterization and tumorigenicity tests. It was observed that the GFP^+^ cells isolated from ascites and solid tumors exhibited unlimited proliferative potential; the monoclonal cells were mouse-original, had a cancer cell phenotype and expressed the macrophage marker protein CD68. Thus, in the abdominal tumor model with double-color fluorescent tracer, macrophages recruited by tumor cells not only promoted tumor cell growth, but also exhibited their own canceration. This discovery is significant for the further study of tumor tissue remodeling and the tumor microenvironment.

## Introduction

Macrophages in the body are known to exhibit the effect of immune surveillance on tumor cells (TCs) (1), although it is now considered that this effect occurs only in the early stages of tumor growth. A large number of immune inflammatory cells, including macrophages, do not provide immune surveillance effects in the aggressive stage. They are therefore known as tumor-associated immune cells, and the macrophage, as a notable member of the inflammatory cells, is known as a tumor-associated macrophage (TAM) (2,3). These cells are widely observed in numerous malignant tumors (4–9). TAMs are recruited into the tumor by inflammatory chemokines and play a key role in tumor proliferation and progression (10–14). With respect to neural tumors, Charles *et al* described in detail various cell/molecular phenotypes of TAMs and other inflammatory cells in the brain tumor microenvironment and their promoting effects of TC proliferation, invasion and metastasis, although the study did not mention the issue of canceration of the TAMs themselves (15). Bouvet *et al* used a liver metastasis model of spleen-inoculated colon cancer cells, and demonstrated the synergistic effects of spleen cells in the process of colon cancer cell metastasis and colonization (16). It is well known that the spleen is an innate organ enriched with immune cells, and that spleen cells with original immune surveillance play a similar role to that of TAMs, described above. However, whether spleen cells would themselves develop canceration has again not been elucidated. We were inspired by the study of Bouvet *et al*, who demonstrated that the self-built double-color fluorescent tracer tumor model was beneficial in the discovery of spleen cell-assisted colon cancer metastasis and colonization, and clearly identified a correlation between the TCs and the host. Therefore, we used a similar method, establishing a double-color fluorescent tracer tumor model of red fluorescent protein as a tracer for TCs and green fluorescent protein for host cells (RFP/GFP) (17), and demonstrated that there were always a number of continuous passaging GFP^+^ cells in the transplanted tumor tissues. These tumor tissue cell suspensions were then cultured *in vitro*, where the existence of continuous passaging GFP^+^ cells was also noted. The cells were identified as TAM cancerous cells originating from the transplanted tumor tissues through a series of macrophage-related and cancer phenotype tests, including cancer genetics and cell biology tests.

## Materials and methods

### Establishment of tumor model

Lentiviral vectors (Genechem Chemical Technology Co., Ltd., Shanghai, China) were used to transfect the RFP gene into human SU3 glioma stem/progenitor cells (self-built in the lab) (18), and SU3 cells were obtained with high expression of RFP (SU3-RFP) screened by puromycin. SU3-RFP cells were cultured in high-glucose Dulbecco’s modified Eagle’s medium (DMEM; Gibco-Invitrogen, Life Technologies, Carlsbad, CA, USA) containing 10% fetal bovine serum (Hyclone, Logan, UT, USA), or in DMEM/F12 (Gibco-Invitrogen) containing 20 ng/ml recombinant human basic fibroblast growth factor (PeproTech, Princeton, NJ, USA) and recombinant human epidermal growth factor (PeproTech). SU3-RFP (150 μl) containing 1×10^6^ cells was injected with a micro-syringe directly into the abdominal cavity of the NC-C57BL/6J-GFP nude mice, which were ~6 weeks old and expressing GFP (19). The mice were then bred in an IVC isolation device (Fengshi Laboratory Animal Equipment Co., Ltd., Suzhou, China) according to specific pathogen-free level management requirements. Approximately one month later, when the abdominal circumference of the mice was observed to have increased from the *in vivo* imaging system (Carestream Health, Rochester, NY, USA), the mice were sacrificed, the ascites were obtained, and an abdominal anatomical procedure was performed to obtain the solid invasive-growing tumors. Cryosectioning was also performed on the peritoneal tumors for observation with a laser scanning confocal microscope (Carl Zeiss, Oberkochen, Germany). This study was carried out in strict accordance with the recommendations in the Guide for the Care and Use of Laboratory Animals of the National Institutes of Health. The animal use protocol was reviewed and approved by the Institutional Animal Care and Use Committee (IACUC) of Soochow University.

### Proliferative host cells cloned from tumor model

Ascites were obtained from the tumor-bearing mice and red blood cells were removed. Solid tumor tissue that had invaded into the liver and gastrointestinal wall was obtained and digested with trypsin into a single cell suspension, then the above targets were subcultured and amplified in DMEM (Gibco) containing 10% fetal bovine serum (Hyclone). Then flow cytometry (Beckman Coulter, Miami, FL, USA) was used to separate GFP^+^ cells for continuous cultivation. The limiting dilution method and the capillary method were performed for the monoclonal cell lines. Once the amplification identified cells of single-cell origin, the cells were frozen in liquid nitrogen for future use. Among these, the cell lines originating from the solid tumor on the gastrointestinal wall underwent further study and were named SU3-induced host celiac tumor cells (SU3-ihCTCs).

### Detection of characteristics of SU3-ihCTCs grown in vitro

DMEM medium containing 10% fetal bovine serum was used for the cultivation of SU3-ihCTCs, then the cell growth was observed with an inverted fluorescence microscope (Carl Zeiss). After developing the SU3-ihCTCs on slides, hematoxylin and eosin (H&E) staining was performed and the cell morphology was observed. A total of 1×10^3^ cells (100 μl) were added onto a 96-well plate, and the 3-(4,5-Dimethylthiazol-2-yl)-2,5-diphenyltetrazolium bromide (MTT) method was used to draw the cell growth curve. To determine the colony formation rate the cells in the logarithmic growth phase were digested with 0.25% trypsin, seeded in six-well plates with 100 cells per well and incubated overnight at 37°C and with 5% CO_2_, then the number of adherent cells was calculated. After culturing for a further 6–8 days, cells were fixed with methanol for 10 min and stained with crystal violet for 20 min, then a microscope was used to determine colony counts (a colony was defined as ≥50 cells grown together) for the final calculation of the clone formation rate: clone formation rate = number of colonies/number of seeded cells × 100. This was performed three times for each well.

### Molecular genetic testing of SU3-ihCTCs

The cellular DNA content of SU3-ihCTCs in the logarithmic growth phase was detected by flow cytometry. Following the method of Seabright (20), cell chromosome G-banding analysis was performed. A DNeasy blood and tissue kit (Qiagen GmbH, Hilden, Germany) was used to extract the cell or tissue DNA, and the cell species was identified using the method reported by Parodi *et al* (21). The primers used for polymerase chain reaction (PCR) amplification of the human-specific h-cox1 gene were 5′-TTCGGCGCATGAGCTGGAGTCC and 5′-TAT GCGGGGAAACGCCATATCG, with a PCR product of 228 bp. The primers for the mouse-specific m-cox1 gene were 5′-ATTACAGCCGTACTGCTCCTAT and 5′-CCCAAAGAA TCAGAACAGATGC, with an amplified product of 150 bp. Western blot analysis was performed to detect GFP expression. RIPA cell disruption buffer (Millipore, Billerica, USA) was added to the collected SU3-ihCTCs and SU3-RFP cells and mouse spleen tissue to extract the total protein, and proteins were quantified using the bicinchoninic acid method. Total protein (50 μg) was isolated by 12% SDS-PAGE electrophoresis and transferred to a PVDF membrane, then reacted with rabbit anti-GFP antibody (1:2,000; Abcam, Hong Kong, China) and mouse anti-GAPDH antibody (1:1,000; Sigma, St. Louis, MO, USA), respectively. Following reaction with the corresponding horseradish peroxidase-conjugated secondary antibody, chemiluminescence detection and X-ray film developing were performed. Immunocytochemistry was performed to detect the expression of macrophage-specific marker protein CD68 with rat anti-mouse CD68 monoclonal antibody (1:200; Abcam).

### Tumorigenicity testing of SU3-ihCTCs and RAW264.7 cells

SU3-ihCTCs and the murine macrophage cell line RAW264.7 (Chinese Academy of Seed Cell Bank, Shanghai, China) were inoculated into the abdominal cavity and right forelimb armpit of nude mice at a concentration of 1×10^7^ cells/150 μl, and the tumorigenicity was observed. RAW264.7 belongs to the mouse macrophage cell line established by Raschke *et al* (22), with established tumorigenicity, and acted as a control of macrophage canceration in this experiment.

## Results

### Transfecting effects of SU3-RFP

**Figure 1:**
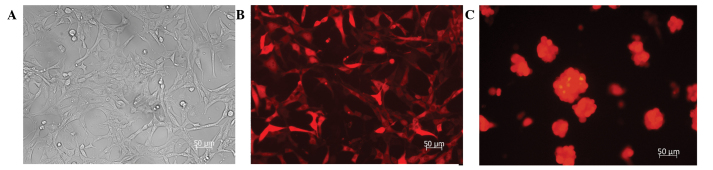
Red fluorescent protein (RFP) gene transfection into SU3 cells (scale bar=50 μm). (A) SU3-RFP cells under inverted microscope; (B) SU3-RFP cells under inverted fluorescence microscope; (C) SU3-RFP suspended cell balls under inverted fluorescence microscope.

The RFP gene was stably transfected into SU3 cells through lentiviral vectors for both differentiated adherent-growing cells in serum culture conditions and suspended-growing stem progenitor cells in growth-factor-containing culture conditions. A red color was observed under the fluorescence microscope due to RFP expression (Fig. 1).

### Double-color fluorescent tracer effects of RFP/GFP tumor model

**Figure 2:**
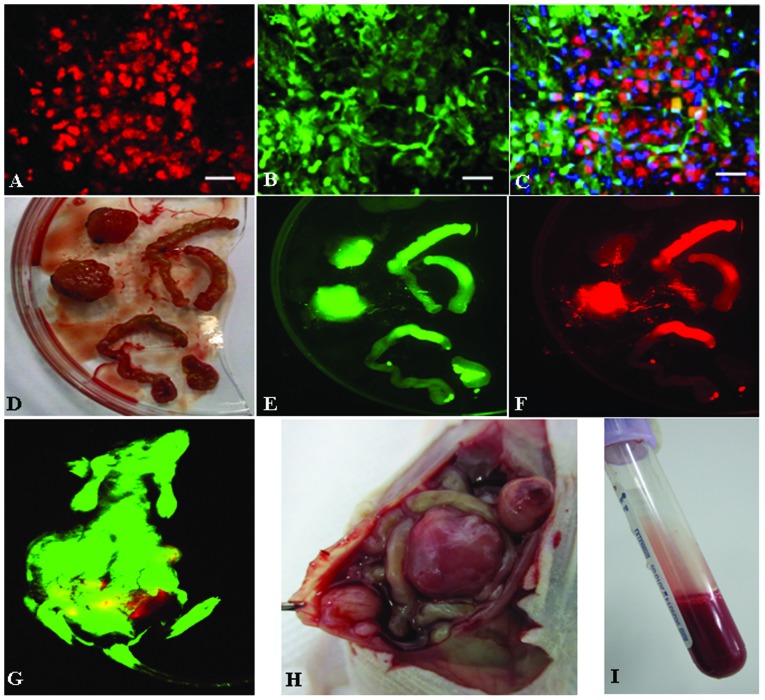
Double-color fluorescent tracer effects of SU3-red fluorescent protein (RFP)/green fluorescent protein (GFP) tumor model. (A–C) Cryosections of abdominal transplanted tumor under confocal microscopy (scale bar=20 μm); (D) Abdominal tumor nodules and the small intestine invaded by tumor cells; (E–F) D in living imaging system; (G) Tumor-bearing mice in living imaging system; (H) Tumor nodules in abdominal anatomy of tumor-bearing mice; (I) Intraperitoneal bloody ascites from tumor-bearing mice.

SU3-RFP cells gave rise to solid tumors (Fig. 2D and H) and malignant ascites (Fig. 2I) in the abdominal cavity of GFP nude mice. In the living tumor-bearing mice (Fig. 2G), in tumor nodules obtained following sacrifice (Fig. 2D–F) and in frozen sections of tumor tissues (Fig. 2A–C), observations revealed that the tumor tissues were composed of the red SU3-RFP fluorescent progenitor cells and the green fluorescent host cells.

### Collection of GFP^+^ cells in transplanted tumors

**Figure 3:**
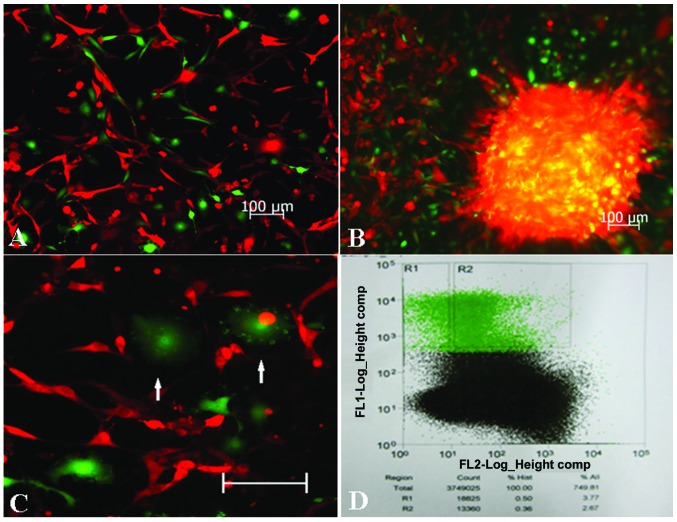
Positive green fluorescent protein (GFP^+^) cells in the transplanted tumor (scale=100 μm). (A) Image of cultivated ascites from SU3-red fluorescent protein (RFP)/green fluorescent protein (GFP) tumor-bearing mice under fluorescence microscope; (B) Image of the primary cultured SU3-RFP/GFP tumor tissue under fluorescence microscope; (C) Macrophages with fluctuating membrane edges (white arrows) in the cultivation of ascites and primary tumor from SU3-RFP/GFP tumor-bearing mice; (D) GFP^+^ cells (R1), accounting for 3.77% of cells in flow cytometry.

The bloody ascites of tumor-bearing mice or tumor nodule tissues were cultured in serum-containing medium, and, while few demonstrated proliferative activity, green fluorescent cells were observed under the fluorescence microscope (Fig. 3A and B). In these green cells, macrophages with a fluctuating membrane edge could be observed (Fig. 3C), among which the majority revealed red fluorescence. Following the digestion of the tumor tissues, GFP^+^ host cells accounted for 3.77% of the mixed cells in flow cytometry (Fig. 3D). The GFP^+^ cells were then separated, enriched with flow cytometry, and the cells could be subcultured continuously.

### In vitro characteristics of SU3-ihCTCs

**Figure 4:**
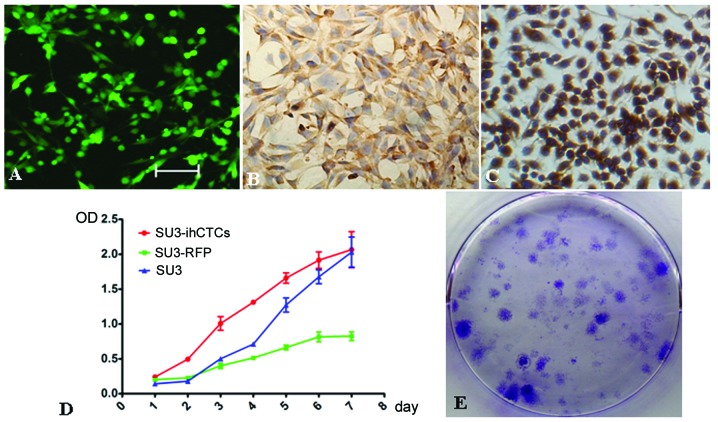
*In vitro* characteristics of SU3-induced host celiac tumor cells (SU3-ihCTCs). (A) SU3-ihCTCs exhibited a green color under the fluorescence microscope (scale=100 μm); (B) Immunocytochemical detection of CD68 expression by SU3-ihCTCs (magnification, ×400); (C) CD68 expressed by RAW264.7 cells (magnification, ×400); (D) MTT determination of growth curves of SU3-ihCTCs, SU3 and SU3-red fluorescent protein (RFP) cells; (E) High cell colony formation rate of SU3-ihCTCs.

Monocloning was performed on the above collected GFP^+^ cells, and unlimited proliferative capacity was confirmed under *in vitro* cultivation. The progenitor cells exhibited green fluorescence during subcultivation. SU3-ihCTCs were pleomorphic, and cell-cell contact inhibition disappeared (Fig. 4A). The growth incubation period and doubling time of SU3-ihCTCs were shorter than those of SU3 and SU3-RFP cells; the cells quickly entered the logarithmic growth phase, exhibiting rapid proliferation in the logarithmic growth phase and a short cell growth cycle (Fig. 4D). The clone formation rates of SU3-ihCTCs and SU3 cells were 57.0±4.4 and 7.3±1.0%, respectively, revealing the capacity of proliferation and formation of a single SU3-ihCTC to be stronger than that of SU3 cells, with low population dependence (Fig. 4E). SU3-ihCTCs highly expressed macrophage-specific marker protein CD68 in the same way as mouse macrophage cell line RAW264.7 (Fig. 4B and C).

### Genetic characteristics of SU3-ihCTCs of murine origin and heteroploidy

**Figure 5:**
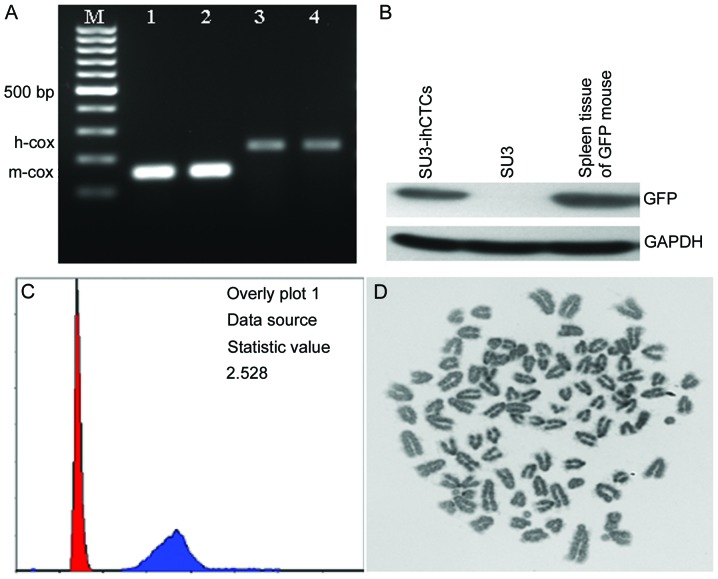
Cytogenetic characterization of SU3-induced host celiac tumor cells (SU3-ihCTCs). (A) Polymerase chain reaction results of cell species. M, 100 bp DNA marker; 1, SU3-ihCTCs; 2, spleen tissue of green fluorescent protein (GFP) nude mouse; 3, SU3 cells for inoculation; 4, normal human peripheral blood lymphocytes. Among these, 1 and 2 amplified mouse cox1 gene positive products (150 bp), 3 and 4 amplified human cox1 gene positive products (228 bp); (B) Western blot analysis results: SU3-ihCTCs and spleen tissues of the GFP nude mouse demonstrated identical GFP expression; (C) Flow cytometry revealed that the DNA synthesis capacity of SU3-ihCTCs was 2.528 times that of lymphocytes; (D) Chromosome G-banding revealed that SU3-ihCTC chromosomes were telocentric, with a median chromosome number of 92.

High cellular DNA synthesis and chromosome changes are major features of TCs. Measured with flow cytometry, the DNA synthesis capacity of SU3-ihCTCs was 2.528 times higher than that of normal diploid mouse lymphocytes, from which it can be assumed that the chromosome number of SU3-ihCTCs was 2.528 times higher than that of normal mouse lymphocytes, and thus that SU3-ihCTCs exceed pentaploidy (2.528×2; Fig. 5C). G-banding calculation was used to calculate the number of chromosomes per cell. In the experiment, the cell chromosome splitting phase was randomly extracted, and the chromosome number was counted to be 92.70±7.15 (n=10) (Fig. 5D). PCR amplification was performed on mouse- and human-specific primers, respectively. Only the mouse cox1 gene was amplified from SU3-ihCTCs (150 bp), and GFP expression was detected using western blot analysis, while the cells revealed features including telocentric chromosomes (Fig. 5C). The above results proved that SU3-ihCTCs were host-derived TCs.

### High tumorigenicity of SU3-ihCTCs

**Figure 6:**
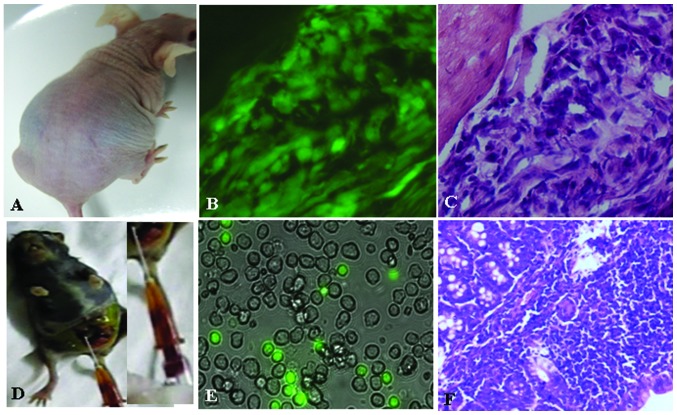
Tumorigenicity tests in SU3-induced host celiac tumor cells (SU3-ihCTCs). (A) SU3-ihCTCs were transplanted into the abdomen of BALB/c nude mice, leading to malignant ascites and abdominal distension; (B) Fluorescence microscope image of cryosections of SU3-ihCTC abdominal implantation (magnification, ×400); (C) Hematoxylin and eosin (H&E) staining of tissue adjacent to B (magnification, ×400); tumor cells exhibited significant pleomorphism and were densely arranged; (D) RAW264.7 was inoculated into green fluorescent protein (GFP) nude mice intraperitoneally, forming bloody ascites and tumor nodules; (E) Fluorescence microscope image (magnification, ×400), RAW264.7-derived cells and host-derived GFP-expressing green-colored cells were observed in the ascites; (F) Optical microscope image of sections of GFP nude mice with RAW264.7 intraperitoneal inoculation (H&E staining, ×100).

In intraperitoneally or subcutaneously inoculated nude mice, the tumorigenicity rate of SU3-ihCTCs was 100% (5/5); ascites and tumor nodules were also formed when SU3-ihCTCs were inoculated into the abdominal cavity (Fig. 6A). Since SU3-ihCTCs were deliberately inoculated in no-fluorescence nude mice, the grown TCs, either ascites or solid tumors, expressed GFP (Fig. 6B); therefore, it could be determined that the TCs were daughter cells of SU3-ihCTCs. The peritoneal solid tumors exhibited clear characteristics of pleomorphism following H&E staining, with a dense arrangement (Fig. 6C). RAW264.7 cells were also inoculated into the abdominal cavity and subcutaneous part of the GFP nude mice, with tumorigenicity rates of 5/5 and 6/6 (Fig. 6D). As the cells were not transfected with fluorescence protein, there was a clear distinction between the GFP^−^ RAW264.7 daughter cells and the GFP^+^ host cells in cultures of the bloody ascites (Fig. 6E). The peritoneal tumor nodules appeared mainly as densely arranged small round cells, and fat vacuoles were also observed (Fig. 6F).

## Discussion

During the progressive stage of malignant tumors, the location of macrophages which play a key role in the innate immune response system has aroused widespread debate in the field of oncology (11–14). Charles *et al* (15) observed that macrophages not only exist in malignant glioma, but also participate in the formation of tumor vessels. This idea contradicts the widespread ‘tumor immune escape’ theory. For tumor immune escape, the TCs escape from the attack of immune cells, and continue to grow. This growth is caused by TCs themselves, and does not involve immune cells. In addition, this theory suggests that the immune cells attacking tumors are differentiated functional cells. Once they enter the senescence or apoptosis stage, the TC attacking ability is lost. The escape of TCs from immune cell attack is due to the insufficient number of immune cells. In contrast, our hypothesis is that immune cells without attacking function do not fade or die, but continue to proliferate under the induction of TCs. At the same time, certain immune cells transform into cancer cells with unlimited proliferative capacity in the same way as TCs. This has not been noted in the study of Charles *et al* (15) or any other publication. It is a novel discovery in a transplanted tumor model using an RFP/GFP double-color fluorescent tracer (Fig. 2).

In this RFP/GFP double-color fluorescent tracer model, the transplanted tumor tissue is cultivated, and one immortalized cell line expressing GFP, named SU3-ihCTC, is cloned (Fig. 4). It is difficult to distinguish between the tumor and host in the traditional non-fluorescence tracer model. At the same time, only the mouse cox1 gene is amplified from SU3-ihCTCs. The DNA synthesis ability detected by flow cytometry and the chromosome G-banding result demonstrate that these cells are heteroploid TCs (Fig. 5) with high tumorigenicity (Fig. 6). Therefore, SU3-ihCTCs are cancerous host cells. Considering the evidence-based medicine level, this can be regarded as level-one evidence. However, the type of host cells from which these cells originate still needs to be confirmed. As observed from the transplanted tumor tissue structure in the double-color fluorescent tracer model (Fig. 2), cells expressing GFP belong to the stromal cell group, which are divided into two categories (cells having parasitic TC tissue, and bone marrow-derived or external circulating cells recruited by TCs) (23). As SU3-ihCTCs are from abdomen-transplanted tumors, they are likely to be peritoneal macrophage-derived cells. Considering that the cells express macrophage marker protein CD68, it is likely that SU3-ihCTCs are macrophage-derived cancer cells. This has been identified from the level of cellular immune marker protein (24). According to a study by Mossor (25), TAMs are divided into two types: typical type (M1) and alternative type (M2). M2 macrophages highly express CD163 and CD204, and their number is positively correlated with the degree of tumor malignancy, playing a role in promoting tumor growth. M1 macrophages are correlated with the immune inflammatory response, demonstrating an anticancer effect. Mora and Regnier-Vigouroux (26) identified that, under induction of lipopolysaccharide, M1 macrophages secrete tumor necrosis factor and induce the apoptosis of M2 cells. Wu *et al* (27) and Waldron *et al* (28) have preliminarily illustrated the molecular mechanism of the transformation of M1 cells to M2 cells and proposed that the transformation is associated with the activation of the STAT3 and PI3K-Akt-mTOR signal transduction pathways. All of these should be confirmed in future studies, in particular the identification of the molecular phenotype and molecular regulation mechanism of SU3-ihCTC and M2 macrophages.

Studies of malignant transformation of stromal cells in human cancer xenografts in nude mice date back to the 1980s (29–37). Goldenberg and Pavia (29) reported that, following *in vitro* short-term subculture, a malignant transformation of host stromal cells is observed in transplanted human cancer in nude mice, but no similar phenomena have been observed in established cell lines. It is speculated that this malignant transformation is induced by a stromal component and not the TCs for inoculation in human cancer tissue. In the present study, the SU3 cells have no stromal component. They are cloned from human malignant glioma tissue with high CD133/nestin expression. Following transplantation into the brain, abdominal cavity and subcutaneous tissue in nude mice, malignant-transformed mouse-derived stromal cells are obtained (SU3-ihBTC, SU3-ihCTC and SU3-ihSTC monoclonal cells, respectively). Therefore, we question the proposal of Goldenberg and Pavia. A significant difference is that the SU3 cells used in our study were stem progenitor cells of malignant glioma, and not traditional TCs cultured *in vitro,* as used in the study of Goldenberg and Pavia. We therefore speculate that in transplanted tumors in nude mice, the initiation factor inducing transformation of murine stromal cells reside in tumor stem progenitor cells for inoculation, and not the original tumor stroma. Of course, this speculation still needs to be confirmed by inoculation of different types of cancer stem progenitor cells to nude mice, which will be performed in the future.

It is naturally essential to clarify the promoter factor inducing the malignant transformation of stromal cells in tumors. Several factors have been considered in previous studies, including horizontal transmission of malignancy by cell fusion (38–42), exosomes (or microvesicles) (43,44) or even sera (45,46). However, more significant is the fact that, if the change in stromal cells in the development of tumors in patients is consistent with the transplanted tumor model, the results of this study have added a new basis to the theory that malignant tumors have heterogeneity (47), with practical value in research on resistance to radiation and chemotherapy of heterogeneous tumors (48). Whether the TCs are of monoclonal or polyclonal origin is still unknown. Goldenberg and Pavia (29) support the latter. We are of the opinion that the initiating cells of the transplanted tumor have gone through the whole process from normal cell to cancer cell, and there is no final conclusion on the origin of the TCs. Recently, Xu *et al* (49) and Hou *et al* (50) applied a single-cell exome sequencing method and established that renal cell carcinoma and leukemia cells are monoclonal-originated. This will be of great assistance in our future research on this topic.
